# Combined deconstructive and reconstructive treatment of a giant vertebrobasilar fenestration aneurysm

**DOI:** 10.3171/2022.7.FOCVID2256

**Published:** 2022-10-01

**Authors:** Lorenzo Rinaldo, Soliman Oushy, Giuseppe Lanzino

**Affiliations:** Departments of 1 Neurosurgery and; 2 Radiology, Mayo Clinic, Rochester, Minnesota

**Keywords:** basilar artery, endovascular procedures, fusiform aneurysm, intracranial aneurysm, vertebral artery

## Abstract

Aneurysms associated with a vertebrobasilar fenestration are rare lesions and can grow to a giant size, presenting significant therapeutic challenges. Endovascular treatment of these aneurysms has traditionally been with coiling; however, flow diverter placement within the fenestration arms has recently proven to be a viable treatment strategy. The authors present a case of a giant vertebrobasilar fenestration aneurysm in a patient presenting with a cranial nerve VI palsy. The lesion was treated by using a combination of flow diverter placement and vertebral artery sacrifice. The nuances of flow diversion therapy for these aneurysms and the management of intra- and postoperative complications are discussed.

The video can be found here: https://stream.cadmore.media/r10.3171/2022.7.FOCVID2256

## Transcript

In this video we present a case of a giant vertebrobasilar fenestration aneurysm treated using a combination of flow diversion and vertebral artery sacrifice.

Our patient is a 47-year-old female who presented to our institution 3 months after the sudden onset of double vision. She denied any other symptoms. Her past medical history was notable for hypertension, and she was an active cigarette smoker with a 30 pack-year history. She had a family history of subarachnoid hemorrhage in her father and aortic aneurysms in her mother and sister. Her physical exam was remarkable for a right cranial nerve VI palsy.

Shown here are T1-weighted gadolinium-enhanced images demonstrating a giant aneurysm originating from the vertebrobasilar junction, indicated by the arrow, with significant mass effect on the medulla and pons. Despite the mass effect, there was no significant edema surrounding the lesion as seen on T2-weighted images. 3D reconstructions of CT angiography showed a bilobed aneurysm associated with a vertebrobasilar fenestration, the arms of which were marked by the arrow.

The patient was taken for formal angiography, which demonstrated significant stasis of contrast within the larger aneurysm sac well into the venous phase. 3D rotational angiography was performed, and on reconstructions the separate arms of the fenestration are again demonstrated.

### 2:03 Treatment.

Given the patient’s young age and the aneurysm’s giant size and symptomatic status, we recommended treatment. Vertebrobasilar fenestration aneurysms are very rare, and thus standardized treatment strategies are lacking. Flow diverter placement within the fenestration arms with or without coiling of the aneurysm sac has been shown to be effective in a small case series,^1^ and this was our favored strategy. Alternative strategies include coiling with or without stent placement within the fenestration arms, which has also been shown to be successful in case reports and small case series.^2–5^ Surgical treatment would entail aneurysm trapping or proximal vertebral artery sacrifice with or without bypass to the posterior cerebral artery depending on the patient’s collateral status, which we would consider in the event of endovascular failure.

### 2:42 Flow Diverter Placement.

We elected to proceed with flow diverter placement. The patient was treated with aspirin and clopidogrel starting 5 days prior to the procedure. She was placed under general anesthesia and bilateral femoral access was secured. The patient was fully heparinized with an activated clotting time maintained above 250 for the entirety of the procedure. A 6-Fr guide catheter was inserted directly into bilateral femoral arteries and navigated to the ostium of both right and left vertebral arteries. A 6-Fr intermediate catheter was brought to the intracranial vertebral artery, and an 0.027-inch microcatheter was navigated distal to the aneurysm.

Here you can see the microcatheter navigated to the right intracranial vertebral artery. Due to the unclear location of the fenestration arms, we experienced significant difficulty traversing the aneurysm, though we were eventually able to do so. However, the microcatheter would initially not track into the basilar artery, as shown here.

### 3:38 Traversing Right Fenestration Arm.

To improve the step-off, we passed an 0.010-inch microwire, or "buddy" wire, through the intermediate catheter, which eventually facilitated passage of the microcatheter into the basilar artery. We then traversed the left-sided fenestration and navigated a second 0.027-inch microcatheter across the aneurysm and into the basilar artery.

### 4:19 Left Flow Diverter Deployment.

We then started to deploy a 3 × 18–mm Pipeline embolization device through the left-sided microcatheter. We aimed to land the distal end of the device just distal to the fenestration arm and flaring into the basilar artery.

Unsubtracted images showed the device to be opening with the distal end in the desired location, and the device was fully subsequently deployed. Fluoroscopy confirmed good position of the left-sided device with adequate wall apposition as seen on angiography.

### 5:19 Right Flow Diverter Deployment.

We then began deploying a 3 × 18–mm Pipeline embolization device in the right fenestration arm; however, as we pushed the device the distal end appeared to fall into the aneurysm, which was confirmed on unsubtracted images. The arrow marks the location of the distal end of the right-sided device, which appears to be within the aneurysm.

We planned to resheath the device and subsequently redeploy it more distally; however, despite repeated efforts we were unable to bring the device back into the microcatheter. We considered removing the device partially unsheathed; however, we were concerned about the possibility of thromboembolic events during this process. We therefore decided to deploy the right-sided device in suboptimal position and proceed with right vertebral artery sacrifice. We subsequently navigated a coiling microcatheter through the right-sided device and into the larger aneurysm sac.

### 6:43 Aneurysm Coiling.

The larger aneurysm lobe was loosely coiled with two 14 × 40 helical coils in order to lessen the amount of intra-aneurysmal thrombus formation, potentially lowering the likelihood of acute aneurysm rupture after treatment.

### 7:01 Microvascular Plug Placement.

We then deposited a microvascular plug at the vertebrobasilar junction distal to the origin of PICA. Following placement of the plug, flow through the right vertebral artery was significantly diminished. We then placed a final 1.5 × 4 helical coil, again distal to the origin of PICA.

Repeat right vertebral artery angiography demonstrated essentially no flow into the aneurysm from the right side. Left-sided vertebral angiography already demonstrated reduced aneurysm filling and no evidence of thromboembolic complications.

To avoid fulminant aneurysm thrombosis and possibly rupture, an abciximab infusion was started at the end of the procedure and continued overnight. The patient awoke at her baseline neurological condition with a stable right cranial nerve VI palsy and no other deficits.

### 8:10 Postoperative MRA.

An MRA obtained on postoperative day 1 demonstrated patency of the left vertebral and basilar artery with significantly reduced filling of the aneurysm. DWI sequences demonstrated a small focus of infarction within the left pons that was fortunately asymptomatic.

The patient had an uneventful recovery and was discharged on postoperative day 2 with a plan to continue aspirin and clopidogrel until follow-up. Approximately 1 month after discharge she developed worsened double vision and new left-sided hemiparesis.

### 8:48 One-Month MRI.

A repeat MRI was obtained that demonstrated interval development of significant perianeurysmal edema, presumably secondary to aneurysm thrombosis. She was started on a slow steroid taper and her symptoms gradually resolved. An MRI obtained 9 months after treatment demonstrated resolution of the edema and contraction of the thrombosed aneurysm. Repeat angiography performed 1 year after treatment demonstrated only a small amount of residual aneurysm filling. The right vertebral artery remained occluded past the origin of PICA.

### 9:38 Final Follow-Up MRA.

An MRA obtained 5 years after treatment demonstrated continued patency of the left vertebral and basilar artery with no residual aneurysm filling. Clinically, the patient remains with the right-sided cranial nerve VI palsy she had on presentation, but with no other symptoms. Thank you.

## Disclosures

Dr. Lanzino: consultant for Superior Medical Editors and Nested Knowledge.

## Author Contributions

Primary surgeon: Lanzino. Assistant surgeon: Rinaldo. Editing and drafting the video and abstract: Rinaldo, Oushy. Critically revising the work: Rinaldo, Lanzino. Reviewed submitted version of the work: all authors. Approved the final version of the work on behalf of all authors: Rinaldo. Supervision: Lanzino.
